# Treatment of Fragile X Syndrome with Cannabidiol: A Case Series Study and Brief Review of the Literature

**DOI:** 10.1089/can.2018.0053

**Published:** 2019-03-13

**Authors:** Nicole Tartaglia, Marcel Bonn-Miller, Randi Hagerman

**Affiliations:** ^1^ Department of Pediatrics, University of Colorado School of Medicine, Aurora, Colorado.; ^2^ Zynerba Pharmaceuticals, Devon, Pennsylvania.; ^3^ Department of Pediatrics, MIND Institute, University of California Davis Medical Center, Sacramento, California.

**Keywords:** cannabidiol, CBD, endocannabinoid system, fragile X syndrome, oral solution

## Abstract

Fragile X syndrome (FXS) is an X-linked dominant disorder caused by a mutation in the fragile X mental retardation 1 gene. Cannabidiol (CBD) is an exogenous phytocannabinoid with therapeutic potential for individuals with anxiety, poor sleep, and cognitive deficits, as well as populations with endocannabinoid deficiencies, such as those who suffer from FXS. The objective of this study was to provide a brief narrative review of recent literature on endocannabinoids and FXS and to present a case series describing three patients with FXS who were treated with oral CBD-enriched (CBD+) solutions. We review recent animal and human studies of endocannabinoids in FXS and present the cases of one child and two adults with FXS who were treated with various oral botanical CBD+ solutions delivering doses of 32.0 to 63.9 mg daily. Multiple experimental and clinical models of FXS combine to highlight the therapeutic potential of CBD for management of FXS. All three patients described in the case series exhibited functional benefit following the use of oral CBD+ solutions, including noticeable reductions in social avoidance and anxiety, as well as improvements in sleep, feeding, motor coordination, language skills, anxiety, and sensory processing. Two of the described patients exhibited a reemergence of a number of FXS symptoms following cessation of CBD+ treatment (e.g., anxiety), which then improved again after reintroduction of CBD+ treatment. Findings highlight the importance of exploring the therapeutic potential of CBD within the context of rigorous clinical trials.

## Introduction

Fragile X syndrome (FXS) is an X-linked dominant disorder caused by the expansion of a trinucleotide repeat (CGG)n within the first exon of the fragile X mental retardation 1 (*FMR1*) gene, which silences the expression of the fragile X mental retardation protein (FMRP).^1^ The absence of FMRP, an important regulator of translation of many messenger RNAs involved in synaptic plasticity,^2^ leads to substantial intellectual deficits. The condition has been estimated to affect ∼1:4000 males and 1:7000 females.^3–5^ Although both sexes are susceptible, males with FXS typically exhibit more severe symptoms compared with females because the single X chromosome in males is usually fully methylated and not producing FMRP^6^; females typically have some FMRP expression from the *FMR1* gene on their second unaffected X chromosome.^7^ Males with FXS are far more likely than females to exhibit significant intellectual and developmental disabilities (85% vs. 25%).^8^

Among individuals with the full mutation (i.e., >200 CGG repeats), symptoms of FXS vary by age and sex, but often include anxiety (80%), social avoidance (80%), stereotyped behaviors (80%), attention-deficit/hyperactivity disorder (70%), autism spectrum disorders (60%), intellectual disability (85% of males and 25% of females), aggression (40%), disrupted sleep patterns (40%), and epilepsy (16%), as well as macroorchidism (90% of adults), prominent ears (60%), long faces (60%), soft skin (50%), and hyperextensible joints (60% of children).^9–11^ Traditional allopathic treatment of these patients may involve medications to address issues with sleep (melatonin and clonidine), anxiety (selective serotonin reuptake inhibitors and benzodiazepines), hyperactivity and deficits in attention (psychostimulants), and seizures (carbamazepine, valproic acid, and lamotrigine).^12^ Yet, for many patients with FXS, the aforementioned pharmacotherapies, when used alone or in combination, have suboptimal efficacy and tolerability,^11–13^ suggesting a persistent unmet medical need for novel, interventional treatment approaches for patients with FXS. Because the clinical abnormalities in these patients (and most recently in children with autism spectrum disorder^14^) have been linked, at least in part, with dysregulation of the endocannabinoid system, we briefly review recent research characterizing the involvement of the endocannabinoid system in FXS and present the cases of three individuals (one child and two adults) with FXS who experienced functional benefit after treatment with cannabidiol (CBD)-enriched (CBD+) preparations.

## Endocannabinoids and FXS

The endocannabinoid system consists of receptors in the brain and peripheral tissues that are involved in numerous physiological processes as well as the endocannabinoids, N-arachidonoylethanolamine (anandamide; AEA) and 2-arachidonoylglycerol (2-AG). The endocannabinoids bind to the G-protein-coupled receptors, cannabinoid 1 and 2 (CB_1_ and CB_2_),^15,16^ and modulate synaptic transmission throughout the central nervous system.^17,18^ Receptors are distributed throughout the body, with CB_1_ receptors abundantly expressed in the brain and present at lower concentrations in a variety of peripheral tissues and cells and CB_2_ receptors expressed primarily in the immune and hematopoietic systems, as well as in the brain, pancreas, and bone.^16^

CBD, the primary, noneuphoric exogenous phytocannabinoid in cannabis, may attenuate the loss of endogenous cannabinoid (i.e., endocannabinoid) signaling observed in preclinical models of FXS, allowing a component of the FMRP deficiency inherent in FXS to be bypassed. Specifically, many abnormalities seen in FXS appear to be rooted in dysregulation of the endocannabinoid pathways in the central nervous system, with a reduction of endogenous stimulation of endocannabinoid receptors.^19–21^ CBD has the capacity to interact with an FXS-compromised endocannabinoid system. Indeed, deletion of FMRP within a mouse model of FXS led to reduced production of 2-AG, decreasing activation of CB_1_ receptors in the central nervous system.^20^ CBD has been shown to increase 2-AG availability,^22^ potentially attenuating or reversing one of the biological mechanisms of abnormal cellular function in FXS.^20^ Importantly, CB_1_ protein expression appears unaffected in *FMR1* knockout (KO) mice, suggesting that the downstream elements of endocannabinoid signaling can be engaged, even in the absence of FMRP.^19^

In addition to the role of 2-AG, recent work has begun to highlight the potential importance of AEA in addressing social impairment as well as deficits in learning and memory among those with FXS. In an *FMR1* KO mouse model of FXS, Qin et al. demonstrated that increased levels of AEA were associated with greater cognitive performance.^23^ Similarly, Wei et al. utilized mouse models of FXS to show that AEA-mediated signaling at CB_1_ receptors, driven by oxytocin, controls social reward^24^ and that increasing AEA activity resulted in reductions in social impairment.^25^ Much like its impact on 2-AG, CBD has been shown to increase levels of AEA by binding to fatty acid-binding proteins, which transport AEA to the catabolic enzyme fatty acid amide hydrolase, an enzyme that breaks down AEA.^26–28^ Binding to fatty acid-binding proteins is thought to increase AEA availability and CB_1_ activation.

## CBD and FXS: Other Mechanisms of Action

The mechanisms underlying the potential benefits of CBD for FXS span far beyond the endocannabinoid system. Beyond providing benefit to patients with FXS through increases in 2-AG and AEA availability, CBD may positively affect synaptic plasticity. Studies of *FMR1* KO mice have identified discrete alterations in synaptic plasticity in specific brain regions, including an increase in long-term depression (LTD) in the hippocampus,^23,29^ and preclinical data suggest that reducing LTD in *FMR1* KO mice requires activation of endocannabinoid receptors.^19^ Therefore, it is hypothesized that CBD may increase synaptic plasticity in FXS, facilitating one of the basic cellular mechanisms thought to be associated with learning and improvements in cognition.^29^

More recent work has also begun to identify deficits in GABA receptor expression among those with FXS. As FMRP has been shown in animal models to enhance expression of GABA receptors, the lack of FMRP among those with FXS has been associated with fewer GABA receptors.^30^ Indeed, preclinical studies in *FMR1* KO mice have consistently shown downregulation of the GABA system.^11^ As CBD acts as a positive allosteric modulator of GABA-A receptors,^31^ CBD may also act to enhance the binding affinity for GABA.

The impact of CBD on serotonin represents a mechanism by which CBD may aid in reduction of social anxiety and resulting avoidance experienced by patients with FXS. Indeed, the anxiolytic effects of CBD have been reported in over 30 preclinical studies, using multiple models of anxiety (i.e., generalized anxiety, stress-induced anxiety, panic disorder, and contextual fear conditioning),^32,33^ as well as in a growing number of human studies, including within-social anxiety.^32,34^ Several preclinical studies have identified the serotonin 1A receptor as one mechanism through which CBD exerts its anxiolytic effects.^35,36^

## Case Reports

We report the cases of three patients with diagnosed FXS who received oral forms of botanically derived CBD+ solutions and reported clinical symptom improvement.

### Patient 1

Patient 1 is a 3-year 6-month-old male with FXS diagnosed at age 15 months with 250 to 650 CGG repeats. He was born full-term following an uncomplicated pregnancy and delivery at seven pounds six ounces. In the newborn period, he began having problems latching for breastfeeding, and he was fed pumped breast milk and formula from a bottle; he had frequent gagging and spitting up. By 6 months of age, with introduction of soft baby foods, he was often dysphagic, choked easily, and was intolerant of chunky or textured foods. Testing for food allergies was negative, and his formula was changed multiple times over his first year of life. He grew slowly at the third to fifth percentile for weight and the 50th percentile for length until 15 months of age. He was also hypotonic with delays in gross motor skills. He had behavioral concerns, including atypical motor movements, frequent repetitive moving, stiffening and shaking of his legs, body rocking, and repetitive finger stereotypies while touching his ears. He displayed difficulty adjusting to new or noisy places and changes in routine, as well as trouble making eye contact and a short attention span during play and in social interactions; he would sometimes stare off and seem disconnected for 10 to 30 sec.

At 15 months of age, he was evaluated and underwent a brain magnetic resonance imaging (MRI), electroencephalography (EEG), creatine kinase, plasma amino acids, and thyroid and genetic testing. His MRI, EEG, microarray, and laboratory parameters were normal. His FXS DNA test results showed a full mutation for FXS with 260 to 650 fully methylated CGG repeats.

At this time, the patient's parents independently obtained and began administering an oral paste comprising 18% to 23.5% CBD and trace amounts (0.03%) of delta-9-tetrahydrocannabinol (THC) that delivered 50 mg of CBD per day (RSHO™ Blue Label; Naturewell, Inc., San Diego, CA). They combined the CBD paste with coconut oil, heated the mixture until it liquefied, and then used a syringe to administer the liquid preparation orally. During the first month of CBD+ treatment, the family noticed behavioral improvements.

After 1 month of CBD+ monotherapy, the then 16-month-old patient began specialized care in the form of speech and language therapy and occupational therapy for FXS. A baseline measure of adaptive functioning was obtained (Table 1). Over the next 3 months of daily 50-mg CBD+ treatment with adjunctive therapy, the family noted continued improvements in a wide range of clinical parameters. Feeding improved markedly with increased willingness to eat solid foods and increased intake overall. His parents also noted improvements in motor coordination, more frequent vocalizations, less rocking and kicking during feeds, more frequent and longer eye contact, an increase in positive interactions with other children, greater willingness to explore new places, and less self-stimulatory behavior. Many of the parental observations were also confirmed by the patient's occupational and speech therapists, some of whom were unaware of the initiation of CBD+ treatment. CBD+ solution was the only medication used in treatment during this time.

**Table 1.** Patient 1: Adaptive Functioning^a^ at 1 Year 4 Months of Age and 3 Years 0 Months of Age

	1 year 4 months		3 years 0 months	
	Standardized score^b^	Percentile	Standardized score^b^	Percentile
Communication	84	14th	87	19th
Daily living skills	71	3rd	77	6th
Socialization	86	18th	79	8th
Motor skills	78	7th	70	2nd
Adaptive behavior composite	76	5th	75	5th

^a^ Vineland adaptive behavior scales, second edition—parent survey.

^b^ Mean±SD=100±15.

SD, standard deviation.

At the 4-month follow-up visit, the patient's weight had increased to the 15th percentile, while his length remained at the 50th percentile. Developmental progress included improved fine and gross motor skills, decreased repetitive rocking, improved social interest/engagement, improved vocalizations, and decreased hyperactivity. The patient began a regimen of sertraline (2.5 mg per day) due to preliminary evidence of improved language and development in young children with FXS treated with sertraline.^37,38^ Subsequent follow-up visits (at 21 and 27 months of age) showed continued improvement in language and developmental skills. The patient started walking and self-feeding independently, exhibited more frequent and varied vocalizations, fewer repetitive and motor behaviors, fewer sensory sensitivities (e.g., tolerated noise and lights of fireworks), and more frequent initiation of joint attention with pointing. Weight increased to the 25th percentile. He continued using CBD+ solution and remained in a strong early intervention program: 1 h per week of occupational, speech, and physical therapy; 6 h per week of Early Start Denver Model behavioral therapy and applied behavioral analysis.

When the patient was 30 months of age, his parents chose to discontinue CBD+ treatment to instead explore minocycline, shown to improve behavioral symptoms in some patients with FXS.^39^ The initiation of 25 mg per day of minocycline coupled with cessation of CBD+ treatment resulted in increased anxiety, more frequent meltdowns, and more difficulty falling and maintaining sleep.

During a clinic visit when the patient was 3 years of age, it was noted that the patient continued to engage and make slow progress, although anxiety, frequent meltdowns, and difficulties with sleep persisted, and increasing challenges with transitions and attention span were observed. The patient's medical treatments included a daily regimen of 4 mg of sertraline and 25 mg of minocycline. He also continued to receive intensive early intervention services (as described above). At 3 years of age, an updated adaptive behavior assessment was obtained (Table 1). After discussion with his family and medical team, the patient was restarted on CBD+ treatment, 50 mg per day. Following reinitiation of CBD+ treatment, his parents noted reductions in anxiety, fewer meltdowns, and improvements in his ability to fall and stay asleep. The patient has now transitioned into a small preschool where he is continuing to do well and has remained on his CBD+ treatment.

### Patient 2

Patient 2 is a 26-year-old male with full-mutation (>200 CGG repeats) FXS and an IQ score in the mid-50s. Despite a medication regimen that included 60 mg of methylphenidate hydrochloride (once daily), 2.5 mg of aripiprazole, 100 mg of sertraline, 200 mg of minocycline, and 0.2 mg of clonidine (at bedtime), the patient experienced significant symptoms of attention-deficit/hyperactivity disorder, including hyperactivity, inattention, and impulsivity. He also suffered from anxiety, which led to avoidant behavior and sleep disturbances, and some significant features of autism, including social avoidance, poor eye contact, perseverative behavior, hand stereotypies, and tactile defensiveness.

Due to ongoing symptomatology, the patient began taking a liquid preparation containing 63.9% CBD, 4% cannabichromene, 3.4% THC, and 0.7% phenylbiguanide, delivered orally by a 1-mL syringe. The patient's parents obtained the solution independent of their physician. Between 0.05 and 0.1 mL (i.e., 32.0–63.9 mg CBD) of the oral solution was delivered in the morning, once per day for 6 weeks, during which time other therapies remained unchanged.

During the first week of treatment with the CBD+ solution, the patient's family noticed that his anxiety level was reduced and he was able to explore and participate in more activities with less social avoidance. His facility with language increased, as shown by greater capacity to engage in longer more meaningful conversations. The quality and duration of his sleep also improved; he no longer awoke and wandered in the middle of the night. His symptoms of anxiety and linguistic skills continued to improve over the 6-week course of treatment with the CBD+ solution. His parents have continued him on a stable dose of 0.1 mL daily now for 2 years and have observed sustained symptom improvement.

### Patient 3

Patient 3 is a 22-year-old female who was diagnosed with FXS at 9 years of age with a full mutation of >200 CGG repeats. She met all of her early milestones appropriately, but at around 3 to 4 years of age, she developed quite significant anxiety and panic attacks. There was mild benefit associated with initiation of psychological therapies, although her mother described difficulties with new settings, social anxiety, frequent negative perseverative thoughts, and ongoing panic attacks that would vary in frequency depending on her routine and social supports.

Starting in the fourth grade, she had mild academic challenges that were supported with a 504 educational plan through the end of high school. Ongoing anxiety was present and trials of St. John's Wort and other supplements were minimally helpful. She did not receive any other medication treatment. She graduated from college with academic supports, with continued periods of significant anxiety symptoms, social withdrawal, and panic attacks. After college, she began work as an educational aide in a public school, with an exacerbation of anxiety due to this transition into adulthood and pressures of increased independence.

At 22 years of age, her parents provided her with a liquid formulation of hemp oil containing ∼43 mg of CBD daily (Charlotte's Web™ Everyday Advanced; CWB Holdings, Inc., Denver, CO), which she began taking. She described feeling calmer, with fewer perseverative worries, and a cessation of panic attacks. This led to more interactions and activities with peers and improved performance at work. Her mother also noted that she became more engaged socially, calmed more easily when frustrated, and was less likely to fixate on negative aspects of various situations. She missed about 1 week of dosing when on a family vacation out of town, and after a few days without treatment, she experienced recurrence of anxiety symptoms and reemergence of panic attacks. These resolved after she was home and able to restart her CBD+ treatment. She is currently working full time and living independently. She has continued CBD+ treatment for two and a half years with sustained therapeutic benefit.

## Discussion

Based on its morbidity and prevalence, its association with a number of co-occurring problems, such as seizures, anxiety, and sleep disturbances,^13^ and the paucity of efficacious therapeutic options, FXS represents an important public health problem.^6^ The present article provides a brief review of recent research that documents the promise of CBD as a therapeutic agent for patients with FXS. Also described are the cases of one pediatric and two adult patients with FXS for whom CBD+ treatment appears to have contributed to positive changes in anxiety and/or language skills, with no observed adverse events.

Before starting CBD+ treatment, Patient 1 experienced heightened symptoms of anxiety, frequent tantrums, and sleep difficulties. Over the first month of CBD+ monotherapy, and subsequent 3 months of CBD+ treatment combined with speech, language, and occupational therapy, the patient made considerable progress with feeding and weight gain, exhibited better oral–motor coordination, had decreased social avoidance and sensory sensitivities, and showed improvements in attention span/engagement, the frequency and severity of atypical motor movements, and general level of hyperactivity. Upon discontinuation of CBD+ treatment, the patient's prior symptoms reemerged. While a strong therapy program and later addition of other medications likely contributed to this patient's overall developmental progress, the temporally related improvements in anxiety, feeding, tantrums, and sleep—evident when CBD+ treatment was initially started as monotherapy and then reinstated following cessation—are compelling support for the benefits of CBD+ treatment for this patient. By maintaining his adaptive functioning scores from 1 to 3 years of age, the patient demonstrated a significant improvement over the characteristic developmental trajectory of young male children with FXS, where a decrease in adaptive functioning scores is typically observed between 2 and 6 years of age.^40^

Patient 2 showed a similarly encouraging response to CBD+ treatment, experiencing reduced anxiety, improved use of language, and better sleep within 1 week of beginning treatment with a CBD+ solution. It is also remarkable that the patient continued to demonstrate symptom improvement over the initial 6-week treatment period, with longer-term follow-up highlighting continued use of CBD+ solution with sustained benefit.

In Patient 3, the use of CBD+ solution was also associated with a positive effect in a higher functioning female with FXS and long-standing anxiety symptoms. Similar to Patient 1, treatment discontinuation was associated with a recurrence of anxiety symptoms, with reinitiation of CBD+ treatment leading to symptom improvement and resulting long-term use.

The present findings, coupled with the available preclinical data, highlight the potential for CBD as an intervention for individuals with FXS. The existing literature combines to demonstrate that CBD may positively impact individuals with FXS through many mechanisms, including the endocannabinoid system, GABA, and serotonin. While a number of drugs have been developed to target specific systems (e.g., GABA agonists), CBD has the potential to yield a multifaceted benefit to individuals with FXS due to its multiple mechanisms of action. CBD has not only been shown to be generally well tolerated relative to other treatments used in this population,^41^ but also numerous studies have documented its benefits in terms of sleep quality,^42,43^ anxiety (including social anxiety),^34,44^ and cognitive impairment^45^—symptoms experienced by the individuals profiled in the present case series.

These data serve as stepping stones upon which proof-of-concept open-label trials should be based. As with many patients, however, those discussed herein used orally administered botanical CBD+ solution that is not regulated by the Food and Drug Administration and, thus, inconsistencies in availability, quality, purity, and labeling^46,47^ make research, interpretations, and clinical recommendations challenging. The present case series is limited by its reliance on manufacturer-reported cannabinoid content as well as the lack of multimethod assessment of patient symptomatology, including clinimetric data. The observed clinical benefit of CBD+ treatment in case studies, particularly with respect to caregiver-reported behavioral outcomes, must also be interpreted with caution given the significant placebo effects that have been documented in clinical trials of CBD.^48^ Only placebo-controlled trials will be able to elucidate the true therapeutic effects of CBD/CBD+ treatment on FXS symptomatology. Until rigorous clinical trials have demonstrated the efficacy of CBD/CBD+ treatment for FXS, current treatments for the many behavioral problems associated with FXS should be utilized before off-label use of CBD+ products.

In an effort to overcome existing limitations, future studies should independently test patient samples to confirm actual constituents of each CBD+ preparation^47^ and utilize well-validated caregiver-reported assessments of anxiety and other FXS symptomatology, in addition to unstructured caregiver- and clinician-based reports, in an attempt to more completely track each patient's course while in clinical care. Due to inconsistencies observed in many oral botanical preparations, rigorous examinations of pharmaceutical-grade preparations of CBD should be explored as potential treatments for children and adolescents with FXS.

## Abbreviations Used

AEA: N-arachidonoylethanolamine (anandamide)
2-AG: 2-arachidonoylglycerol
CBD: cannabidiol
CBD+: CBD-enriched
EEG: electroencephalography
FMR1: fragile X mental retardation 1
FMRP: fragile X mental retardation protein
FXS: fragile X syndrome
KO: knockout
LTD: long-term depression
MRI: magnetic resonance imaging
SD: standard deviation
THC: tetrahydrocannabinol
